# A controlled trial of quetiapine XR coadministration treatment of SSRI-resistant panic disorder

**DOI:** 10.1186/s12991-015-0064-0

**Published:** 2015-09-15

**Authors:** Andrew W. Goddard, Waqar Mahmud, Carla Medlock, Yong-Wook Shin, Anantha Shekhar

**Affiliations:** Indiana University Department of Psychiatry, Indianapolis, IN USA; Department of Psychiatry, Ulsan University School of Medicine, Seoul, Republic of Korea; UCSF-Fresno Department of Psychiatry, Fresno, CA USA

**Keywords:** Panic disorder, Atypical neuroleptics, Quetiapine XR, Augmentation treatment

## Abstract

**Background:**

Open-label quetiapine coadministration with SSRI therapy, in a diagnostically mixed sample of comorbid anxiety patients, offered additional anxiolytic benefit. Therefore, we designed the following controlled trial to confirm these findings in a comorbid, SSRI-resistant, panic disorder (PD) patient sample.

**Methods:**

This was a single-site, double-blind, placebo-controlled (PLAC), randomized, parallel group (2 groups), 8-week, quetiapine extended release (XR) coadministration trial. SSRI resistance was determined either historically or prospectively. Patients were randomized if they remained moderately ill (CGI-S score ≥ 4). Change in the PDSS scale total score was the primary efficacy outcome measure. Responders were identified as those with a ≥50 % decrease from their baseline PDSS score. In the early weeks of therapy, XR was flexibly and gradually titrated from 50 to 400 mg/day.

**Results:**

43 patients were screened in total, and 26 of these were randomized and evaluable. 21 patients (78 % of the randomized group) completed the trial (10 XR; 11 PLAC). The endpoint quetiapine XR mean daily dose ± SD was 150 ± 106 mg. While, in the sample as a whole, there was improvement in PDSS scores across the 8-week trial (ANOVA main effect of time, *F* = 10.9, *df*_8,192_, *p* < 0.0001), the treatment × time interaction effect was not statistically significant (*F* = 0.8, *df*_8,192_, *p* = 0.61). There was no between-group difference in responder frequency at endpoint.

**Conclusions:**

This proof-of-concept RCT did not support the efficacy of this treatment strategy for SSRI-resistant PD. Quetiapine XR was generally well-tolerated. Important limitations were the small sample size, and the relatively low average dose of quetiapine XR used.

ClinicalTrials.gov ID#: NCT00619892

## Background

While panic disorder (PD) is generally considered a treatment-responsive psychiatric disorder, panic patients with agoraphobia and other mood/anxiety disorder comorbidities pose a significant therapeutic challenge, and have a poorer longer-term prognosis than patients with uncomplicated PD [1]. Therefore, clinical studies have begun to evaluate the benefit of coadministration protocols that could improve short and longer-term outcomes in patients with comorbid panic. In earlier work, we observed superior early stabilization of SSRI-treated PD patients (30 % also had major depression) with the co-addition of the benzodiazepine, clonazepam [2]. However, maintenance benzodiazepine therapy can be complicated with psychological and physiological dependence. Other classes of agents with anxiolytic potential, such as the atypical neuroleptics [3, 4], may also be usefully combined with SSRIs to facilitate early improvement in comorbid PD, and could be more appropriate for maintenance treatment. An open-label, 9-week trial (*n* = 11 pts) observed that quetiapine coadministration (mean endpoint dose = 180 mg/day) with ongoing SSRI therapy in comorbid anxiety patients can have anxiolytic benefit within 1–2 weeks of coadministration therapy [5]. Therefore, the purpose of the present study was to conduct a controlled, proof-of-concept trial to confirm these results in a sample of comorbid, SSRI-resistant, PD patients.

## Methods

### Design

The trial was a double-blind, placebo-controlled, randomized, 8-week, quetiapine XR coadministration trial in patients with SSRI-resistant, comorbid PD. In order to model clinical practice, study medications were administered in a flexible-dosing schedule (see Table 1). Patients were randomly assigned either to identically-appearing tablets of quetiapine XR (50–400 mg p.o. at night)(XR) or placebo (PLAC). For patients receiving adequate (8 weeks or longer, in sufficient doses), ongoing SSRI therapy at intake, SSRI resistance was ascertained by psychiatrist’s clinical impression of only minimal improvement (a CGI-I level ≥3) [6] with the current SSRI trial (20/26 (77 %) of evaluable cases). Patients who were medication-free at intake were initially treated for 8 weeks with open-label, sertraline (50–200 mg/day); citalopram (20–40 mg/day) or escitalopram (10–20 mg/day). Following open-label SSRI treatment, patients that had a <50 % decrease from baseline in the PDSS total score after the prospective SSRI trial, were classified as “resistant”. The study protocol was approved by an IUPUI clinical studies IRB committee (study #0703-22), before any patient work was conducted. The progress of the study was monitored annually by the IRB, and bi-annually by the IU Psychiatry Department Adult Studies DSMB committee.

**Table 1:** Baseline patient characteristics (ITT sample; mean ± SD values reported)

Variable	Quetiapine XR (*n* = 13)	Placebo (*n* = 13)	*P* value
% Female	77 (10/13)	62 (8/13)	0.67^a^
Age (years)	35.5 ± 9.6	35.5 ± 16.8	0.99
Total PDSS score	14.8 ± 3.6	13.7 ± 3	0.40
CGI-S score	4.8 ± 0.8	4.4 ± 0.7	0.19
Total HAM-A score	21.2 ± 6.8	17 ± 5.2	0.09
Total HAM-D score	14.6 ± 6.2	11.5 ± 5.1	0.18
PSQI sleep hours	6.2 ± 1.3	6.7 ± 1.6	0.39
PSQI sleep quality	2.9 ± 0.6	2.3 ± 0.6	0.02

^a^Fisher’s exact test. All other baseline comparisons analyzed by independent *t* test

### Subjects

We recruited subjects into the IU Anxiety Research Clinic located at Indiana University Hospital Adult Psychiatry Clinic and Study Center, Indianapolis. We employed a mixture of recruitment strategies including referrals from local clinicians, flyers displayed in the university hospital, on-line bulletins, and paid advertisements in the local newspapers. Participating patients received a small stipend for attendance at each study visit ($15 per visit).

At the initial study visit (Visit 1), and after giving their written, informed consent (IUPUI IRB study #0703-22), patients underwent a comprehensive medical and psychiatric assessment with the following elements: (1) A clinical psychiatric interview, including the Mini International Neuropsychiatric Interview (MINI) [7] (MINI Plus version 5.0), to confirm the PD diagnosis with or without agoraphobia, (2) psychiatric, medical and surgical history-taking, (3) prior and concomitant medication and procedures (past 30 days), (4) a physical exam including vital signs, (5) a 12-lead ECG, (6) clinical chemistry/hematology including: blood for CBC with differential, comprehensive metabolic panel, and thyroid function test (T3, T4, TSH), (7) a urine toxicology screen, (8) a urine pregnancy test for women of childbearing potential, and (9) a urinalysis.

The main study inclusion criteria were as follows: age ≥18 years, having a primary, current DSM-IV TR diagnosis of PD with or without agoraphobia [8]; and a CGI-S score of ≥4 (moderate illness severity) [6]. Secondary (non-principal) anxiety and mood disorders were allowed. Medical comorbidities were allowed provided that medical problems were currently well-controlled. Key exclusion criteria included the following items: lifetime psychotic or bipolar diagnoses; current pregnancy or lactation; patient currently at significant risk for suicide; a substance abuse disorder within 6 months of intake; an unstable medical condition; a history of type I or type II diabetes; and a history of neurological disorder affecting the CNS.

### Prescribing protocol

The dosing range of quetiapine XR we used was 50–400 mg/day. Our target daily dose for quetiapine XR was 200 mg/day. The detailed quetiapine XR dosing guidelines were as follows: 50 mg one tab po at HS × 3 days, then, if 50 mg tolerated, increase to 50 mg 2 tabs at HS × 4 days; at the beginning of week 2, if the last dose was tolerated increase to 50 mg 3 tabs at HS × 3 days, then, if 150 mg tolerated, increase to 4 tabs at HS; at the beginning of week 3, if no efficacy and the 200 mg dose was well tolerated, increase to one 300 mg tab at HS-otherwise remain at 200 mg one tab at HS; at the beginning of week 4 if still no improvement, and 300 mg was tolerable, increase to 200 mg tablet 2 at HS. From the beginning of week 5 to the end of the trial, quetiapine XR doses were held. We used quetiapine XR tablets and identical-appearing PLAC tablets provided by Astra Zeneca (50, 200, and 300 mg designations). The extend release (XR) preparation of quetiapine was chosen for its potential to limit common side-effects such as sedation. Open-label SSRI/SNRI prescriptions were provided by the study psychiatrists (WM, YS, AWG). Baseline SSRI/SNRI doses were held constant throughout the 8-week trial. Participants were randomized sequentially by a private research pharmacy (Custom Med, Indianapolis). The study coordinator (CM)(who was not involved in the administration of patient ratings) interacted with the research pharmacy to obtain appropriate medication bottles for each patient to be randomized and at each follow-up visit. Medication adherence was monitored weekly by the prescribing psychiatrist by clinical inquiry and assessment of medication bottle returns. Summary records of dispensing and returns were maintained in the patient’s hard-copy medical chart.

Prohibited medication during the study included the following: potent cytochrome P450 inhibitors (including but not limited to ketoconazole, itraconazole, fluconazole, erythromycin, clarithromycin, troleandomycin, indinavir, nelfinavir, ritonavir, fluvoxamine and saquinavir), potent cytochrome P450 inducers (including but not limited to phenytoin, carbamazepine, barbiturates, rifampin, St. John’s Wort, and glucocorticoids), benzodiazepines, anticonvulsants (new med starts), other antipsychotics, lithium, non-SSRI/SNRI antidepressants, and buspirone. Patients were free from standing psychiatric medications (except for their ongoing SSRI/SNRI medicine) for 2 weeks prior to the baseline/randomization visit. Occasional PRN use (not more than 3 doses/week) of a short-acting benzodiazepine in the 2 weeks prior to baseline was permitted if clinically necessary.

### Clinical measures

#### Efficacy measures

The primary study objective was to test the hypothesis that SSRI plus quetiapine XR would result in superior early stabilization of SSRI-resistant, comorbid PD patients vs. SSRI/PLAC, as evidenced by more pronounced, clinically significant decreases from baseline in total panic disorder severity scale scores (PDSS) [9]. Accordingly, weekly PDSS assessments were administered by the study psychiatrists. Secondary objectives of the study were to explore whether SSRI/quetiapine XR-treated comorbid PD patients would also have superior outcomes vs SSRI/PLAC patients on standard measures of depressive symptomatology (HAM-D; baseline, week 2, week 4, week 8) [10], generalized anxiety symptomatology (HAM-A; baseline, week 2, week 4, and week 8) [11], sleep hours and quality (PSQI self-report items done weekly) [12], and global measures of illness severity and improvement (Clinician CGI-S and CGI-I; done weekly) [6].

#### Safety assessments

Study psychiatrists reviewed adverse events/side-effects on a weekly basis. They also administered weekly movement side-effect scales, including the Barnes Akathisia rating scale [13], and the Simpson-Angus EPS scale [14]. Satisfactory physical health was ascertained at the screening visit (visit 1) as previously described. In addition, urine toxicology was repeated at visit 8 to monitor for surreptitious use of benzodiazepines. Vital signs including weight were assessed at each visit throughout the 9-week study. Blood glucose levels were tested at screening, midtrial, and at week 8/endpoint.

#### Statistical approach

ANOVA with repeated measures analyses were conducted on continuous measures. If these were significant, post hoc independent *t* tests at each time point were planned to determine the timing of between-group differences. A secondary linear mixed model analysis was also conducted specifically on PDSS data. Non-parametric (Fisher’s exact test) analyses were performed on responder status at the end of weeks 1, 2, 4, and 8/endpoint (evaluating early, mid, and end-trial treatment effects). A “responder” was defined as a patient with ≥50 % improvement from their baseline total PDSS score. Both intent-to-treat (ITT) and completer populations were analyzed. Last-observation-carried-forward imputations were used for the ITT patients who withdrew early. Analyses were performed using SPSS (version 21.0). 2-tailed analytic tests were performed with the threshold for *α* set at 0.05. Our initial power calculations, generated on PDSS data from a published SSRI/PD coadministration trial [2], assumed a large effect size (*d* = 1.0) at weeks 1 or 2, and a need for 15 patients per treatment cell to detect this. Ultimately, only 27/30 planned patients were randomized, due to an administrative decision by the funding company to terminate the study early during the US economic recession.

## Results

### Evaluable patients

The ITT population characteristics at baseline (*n* = 26) were as follows: female gender 17/26 (65 %) males: 9/26 (35 %); the mean ± SD age = 36 ± 13 years.; Caucasian race = 88 % (23/26), African American = 12 % (3/26); mean ± SD PDSS total score = 14 ± 3; the mean CGI-S score = 4.6 ± 0.8; the mean HAM-D score = 13 ± 6, and mean HAM-A score = 19 ± 6. Thus, the patient sample had moderate baseline levels of panic symptomatology. Table 1 compares the baseline clinical/demographic characteristics of each treatment group. For most baseline measures, there were no statistically significant between-group differences. Comorbid conditions: psychiatric comorbidities in the ITT patients included GAD (*n* = 8), PTSD (*n* = 3), major depression single or recurrent (*n* = 8), depression NOS (*n* = 2), dysthymia (*n* = 1), ADD (*n* = 1), and bulimia in partial remission (*n* = 1).

### Medication doses

The mean ± SD endpoint quetiapine XR dose = 150 ± 106 mg. Baseline daily SSRI/SNRI doses were as follows: sertraline (*n* = 7) = 86 ± 24 mg; citalopram (*n* = 7) = 34 ± 10 mg; escitalopram (*n* = 4) = 10 ± 0 mg; fluoxetine (*n* = 2) = 30 ± 0 mg; venlafaxine XR (*n* = 1) = 150 mg; desvenlafaxine (*n* = 1) = 100 mg; fluvoxamine (*n* = 1) = 50 mg; paroxetine (*n* = 2) = 25 ± 0 mg; and duloxetine (*n* = 1) = 120 mg.

### Safety data

**Fig. 1:**
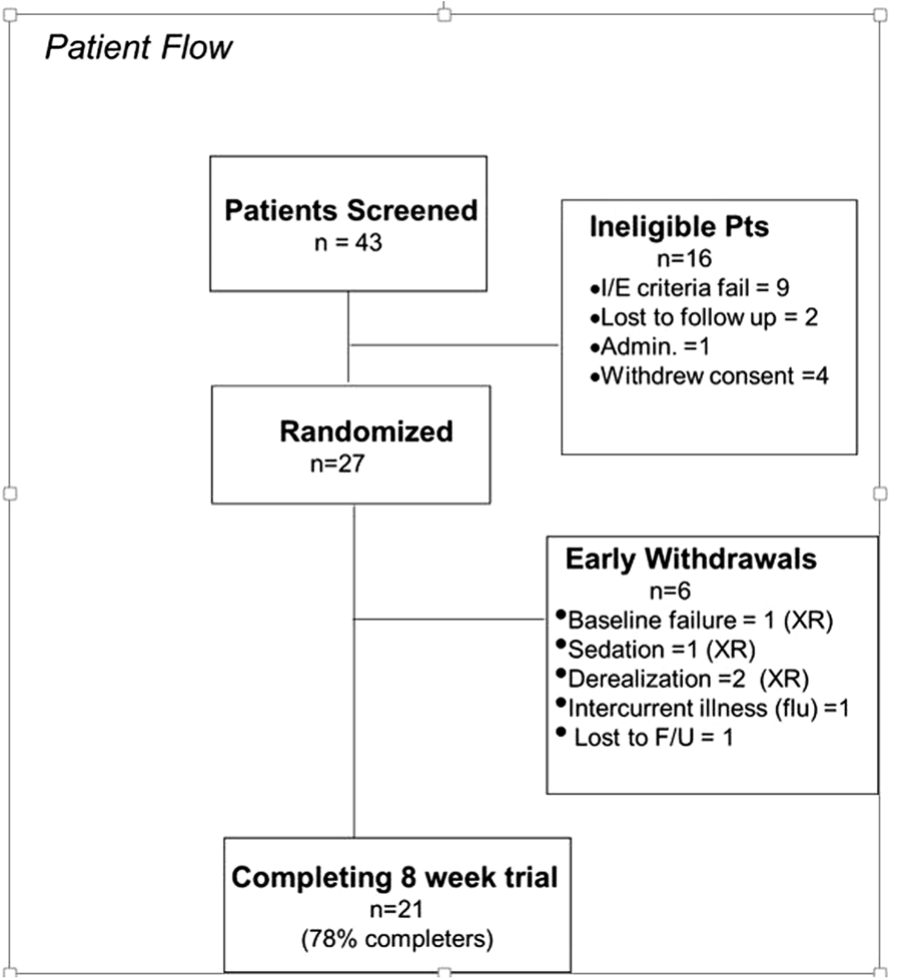
Patient flow summary (randomization: XR *n* = 14, PLAC *n* = 13)

Quetiapine XR was generally well-tolerated with the exception of three patients who discontinued early due to medication-related adverse events (see Fig. 1). Somnolence/sedation was the most commonly reported treatment-emergent adverse event but was usually mild (Table 2); there was no statistical XR/PLAC group difference on frequency of this or other commonly experienced AEs. Vital signs (BP, HR) remained stable during the 8-week trial (data not shown). A slight weight gain was observed in both treatment groups: quetiapine XR (mean ± SD weight; 181 ± 39 to 186 ± 41 lbs at endpoint); PLAC patients (175 ± 50 to 177 ± 48 lbs at endpoint). However, a between-group comparison of weight gains revealed that they were not significantly different (independent *t* test *t* = 1.73, *df* 1, *p* = 0.33) (ITT pts). Blood glucose levels remained within the normal range at screening, midpoint, and endpoint. Urine toxicologies were negative at screening and endpoint. Based on clinical observations, including minimal score changes on the movement symptom scales, neither extrapyramidal side-effects nor akathisia were problematic. For example, mean Simpson Angus total score changes from baseline to endpoint were similar in quetiapine XR and PLAC patients (−0.6 ± 1.0 vs −0.2 ± 0.8; two-tailed *t* = 1.4, *df* = 24, *p* = 0.19). Also, quetiapine XR vs PLAC patients’ mean Barnes Akathisia total score changes from baseline to endpoint were not statistically different (−1.6 ± 1.7 vs −0.5 ± 1.5; *t* = 1.7, *df* = 24, *p* = 0.09).

**Table 2:** Common treatment-emergent adverse events (most to least prevalent)

Adverse event	Quetiapine XR *n* = 13 (*N*, %)	Placebo *n* = 13 (*N*, %)
Somnolence^a^	10 (77 %)	5 (38 %)
Anxiety	4 (31 %)	0 (0 %)
Constipation	3 (23 %)	1 (8 %)
Dry mouth	3 (23 %)	0 (0 %)
Dizziness	2 (15 %)	3 (23 %)
Restlessness	2 (15 %)	4 (31 %)
Derealization^a^	2 (15 %)	0 (0 %)
Insomnia	1 (8 %)	3 (23 %)
Increased appetite	1 (8 %)	2 (15 %)
Leg pain	1 (8 %)	1 (8 %)
Weight loss	1 (8 %)	0 (0 %)
Shakiness	1 (8 %)	0 (0 %)
Muscle aches	0 (0 %)	2 (15 %)
Weight gain	0 (0 %)	1 (8 %)

No significant differences between groups were detected at the *p* < 0.05 level (Fisher’s exact test)

^a^3 patients in the XR group discontinued due to side-effects (somnolence or derealization). There were no serious AEs (ones requiring ER or inpatient care)

## Efficacy data

### PDSS data

**Fig. 2:**
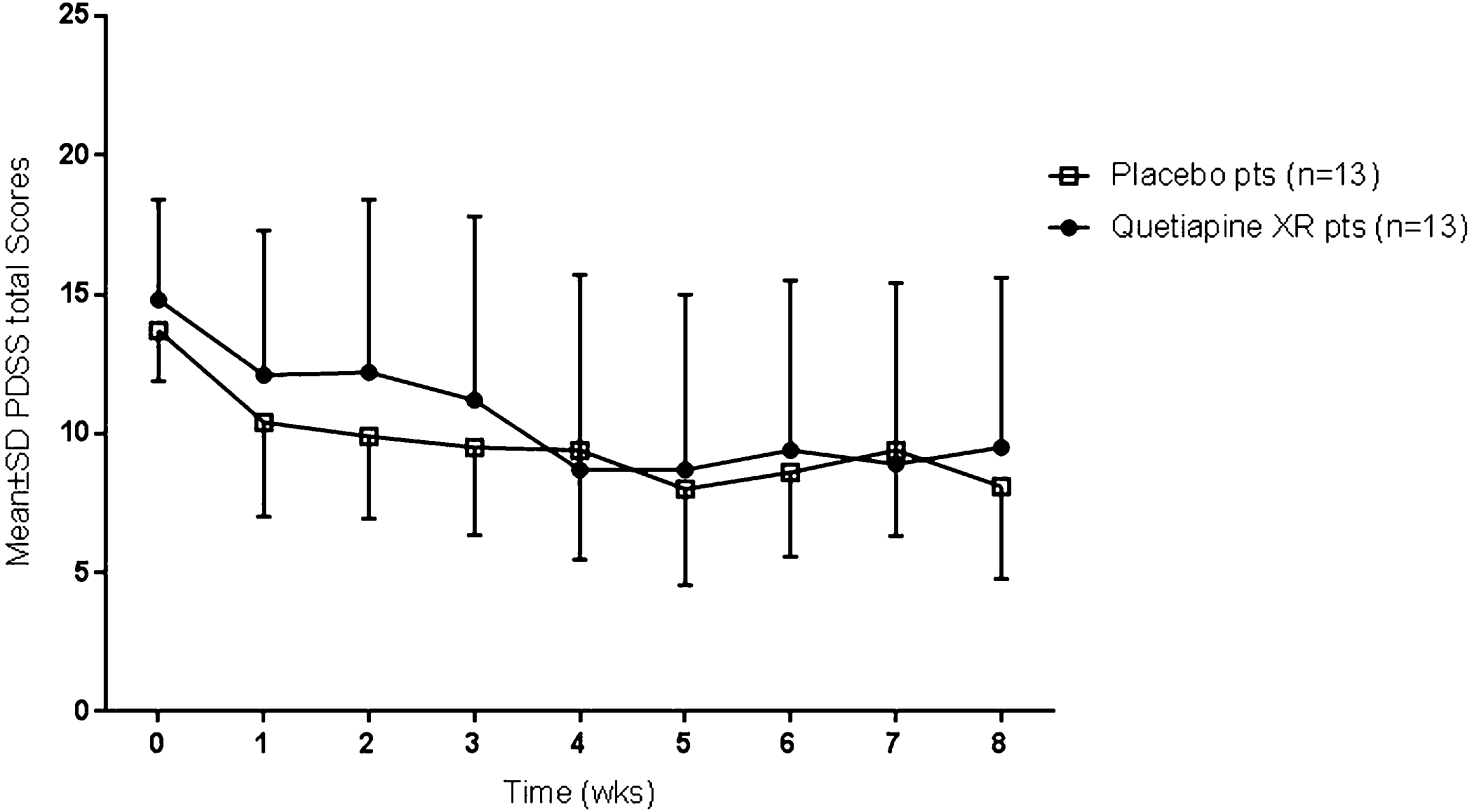
Efficacy data. Mean ± SD PDSS total scores (0–28) (ITT data set; *n* = 26)

The primary efficacy analysis was a repeated measures ANOVA of PDSS total scores data, performed on ITT patients (see Fig. 2 for plot of PDSS scores by time). There was a highly significant main effect of time (*F* = 10.9, *df*_8,192_, *p* < 0.0001), consistent with clinical improvement in the patient sample over the 8-week trial period. However, the treatment × time interaction term was not significant [*F* = 0.8, *df*_8,192_, *p* = 0.61; endpoint mean PDSS difference score = +1.4 (95 % CI −3.1 to 5.9)], indicating that the XR group was not superior to PLAC on this important outcome. Rerunning the analysis using baseline HAM-A scores as a covariate (since there was a trend baseline, between-group difference), did not alter the findings appreciably. A linear mixed models analysis of PDSS scores also failed to detect a drug/placebo difference [fixed effect (active vs placebo), *F* = 0.15; *df*_1,23.9_, *p* = 0.7]. ANOVA of PDSS scores of the completer population, as expected, revealed a highly significant main effect of time (*F* = 12.1, *df*_8,152_, *p* < 0.0001), but a non-significant treatment × time interaction (*F* = 0.63, *df*_8,152_, *p* = 0.75). Furthermore, an ANOVA of the subgroup of patients whose SSRI resistance was historically determined (*n* = 20), also produced similar results. An additional ITT analysis was conducted on item #1 of the PDSS scale (which assesses panic attack frequency/intensity). Again, there was a highly significant main effect of time for this measure (*F* = 7.2, *df*_8,184_, *p* < 0.0001), but a non-significant treatment × time interaction (*F* = 0.51, *df*_8,184_, *p* = 0.85). As mentioned earlier, 11/26 evaluable patients had a comorbid depressive disorder. Therefore, we conducted an exploratory ANOVA to examine the potential impact of depression diagnosis on PDSS total scores over time. The main effect of time was highly significant (*F* = 10, *df*_8,192_, *p* < 0.0001), and there was also a statistically significant depression x time interaction effect (*F* = 2.4, *df*_8,192_, *p* < 0.02), indicating that patients with depression comorbidity (receiving quetiapine or placebo) tended to improve less on the PDSS scale vs non-depressed patients. Notably, though, analyses of the subgroups of non-depressed PD (*n* = 15) and depressed PD patients (*n* = 11), did not reveal significant treatment x time interaction effects (*F* = 0.66, *df*_8,104_, *p* = 0.73, and *F* = 0.81, *df*_8,72_, *p* = 0.6, respectively). Thus, neither presence nor absence of comorbid depression were associated with a tendency to have an improved anti-panic response to quetiapine XR coadministration.

In addition, non-parametric analyses (Fisher’s exact tests) were performed on clinical responder data (ITT sample) at weeks 1, 2, 4, and 8/endpoint, and revealed similar response levels for the 2 treatment groups. At week 1, 1/13 (7 %) of XR patients were classified as responders vs 3/13 (23 %) PLAC patients (*p* = 0.59). After week 2, 2/13 (15 %) quetiapine XR patients were responders vs 3/13 (23 %) PLAC-treated patients (*p* = 1.00). At the end of week 4 (midpoint of the trial), 7/13 (54 %) quetiapine XR cases were responders vs 4/13 (31 %) for PLAC (*p* = 0.43). At endpoint, 6/13 (46 %) of quetiapine XR patients met responder criteria vs 5/13 (38 %) of PLAC patients (*p* = 1.00). Responder analyses performed on the completer patient population produced similar results. We also conducted an analysis on “remitters” at endpoint (patients with a PDSS total score ≤4), and found no between-group difference on this measure (4/13 XR patients vs 3/13 PLAC patients, *p* = 1.00).

### Secondary efficacy measures (CGI-S, CGI-I, HAM-D, HAM-A, PSQI)

Repeated measures ANOVA analyses were also conducted on each of the secondary efficacy measures, with a similar pattern of findings to the primary efficacy analysis (i.e. similar levels of clinical improvement in both groups over the trial period). There was a highly significant main effect of time for the clinician CGI-S measure (*F* = 17.8, *df*_8,192_, *p* < 0.0001), but a non-significant treatment × time interaction (*F* = 0.72, *df*_8,192_, *p* = 0.67). Likewise for the clinician CGI-I outcome measure, there was a highly significant main effect of time (*F* = 6.6, *df*_7,168_, *p* < 0.0001), but a non-significant treatment x time interaction term (*F* = 0.6, *df*_7,168_, *p* = 0.73). HAM-A scores improved over time (main effect of time; *F* = 14.9, *df*_3,72_, *p* < 0.0001), but there was no evidence of superior improvement in the quetiapine XR group (treatment × time interaction *F* = 0.89, *df*_3,72_, *p* = 0.45). Similarly, HAM-D scores improved over time (*F* = 15.4, *df*_3,72_, *p* < 0.0001), but with no significant treatment x time interaction effect (F = 1.9, df_3,72_ p = 0.13). Regarding sleep hours (a PQSI self-report item), there was both a trend level of statistical significance for the ANOVA main effect of time (*F* = 1.94, *df*_8,192_, *p* < 0.06), and for the treatment x time interaction (*F* = 1.85, *df*_8,192_, *p* < 0.07). The latter trend finding was related to early improvement (over the first 4 weeks) in sleep time in the quetiapine XR group. For the sleep quality item of the PSQI, there was a significant main effect of time (*F* = 2.02, *df*_8,192_, *p* < 0.05), but a non-significant treatment x time effect (*F* = 1.1, *df*_8,192_, *p* = 0.36).

## Discussion

In this trial, we did not observe efficacy of the quetiapine XR augmentation strategy on both primary and secondary efficacy measures, in contrast to the positive findings of the case-report/open-label trial literature [4, 5]. There was a trend toward improvement in sleep time in quetiapine XR group, consistent with sleep benefits reported in several recent quetiapine trials [15, 16]. Comorbid depression was associated with relative resistance to treatment (XR or PLAC). Our results supported the patient acceptability and safety of flexible-dose quetiapine XR augmentation for resistant PD. Though sedation/somnolence was commonly reported, other concerning AEs, such as metabolic or extrapyramidal side-effects, were not observed over the relatively brief time-frame of the trial.

This investigation was the first RCT, which we are aware of, that has studied atypical neuroleptic augmentation of SSRI treatment in patients with a primary diagnosis of PD. While there has been a recent trend towards “off-label” prescribing of atypicals for anxiety disorders such as PD [17], there have been few controlled studies to inform this practice. To date, the best evidence supporting atypical use for anxiety syndromes is for generalized anxiety disorder [18–20] and OCD [21]. Of note, however, one GAD trial of atypical augmentation of SSRI therapy, was negative. [22] However, with regard to PD, RCTs studying the antipanic effects of atypical neuroleptics (quetiapine, risperidone, ziprasidone) have primarily evaluated bipolar patients with comorbid panic symptoms [23–25]. Of these trials, only the quetiapine (monotherapy) one was positive.

The strengths of this proof-of-concept trial included the significance for clinical practice (addressing a common clinical dilemma of what to do when SSRI-resistance occurs), the controlled design, the use of psychiatrists to monitor patient safety and administer key efficacy ratings such as the PDSS and CGI ratings, the careful medical screening and patient selection, and the flexible treatment protocol mirroring clinical practice. Limitations of the trial included the small sample size and being underpowered to detect small-moderate effects. To be powered to detect a modest effect, similar to the mid-trial responder results, would have required approximately 50 patients per treatment condition. Additional design limitations included the mixed method of determining SSRI resistance, possible under-dosing of XR in the first 4 weeks (our mean endpoint XR dose = 150 mg (vs 186 mg in the positive quetiapine trial of Sheehan et al. [24]), and the lack of independent evaluators/raters. Relatively low-dosing of quetiapine XR, however, may be preferable in PD and other anxiety spectrum disorders, given that, at the 150 mg/day dose level, optimal anxiolytic effects have been observed in GAD patients [18]. Furthermore, in laboratory models, the putative anxiolytic action of atypical antipsychotic agents (prefrontal cortical 5-HT2A/C receptor antagonism) appears to be optimal at lower dose levels [26, 27]. In addition, though the placebo augmentation response was significant, it is important to note that all subjects were on an active anti-panic regimen (SSRI/SNRI treatment), and a prolonged trial of these medications may well have resulted in a response 8 weeks later. The benefits seen with placebo treatment was also not inconsistent with what is generally reported in panic clinical trials [28, 29]. However, a less frequent visit schedule may have helped to limit this further, thereby improving signal detection. Other design features, such as a placebo run-in period, could also have limited placebo response. Of note, on inspection (Fig. 2) during most of the trial there was increased variability of PDSS scores in the XR vs PLAC-treated patients, possibly reflecting increased anxiety of some patients in response to XR side-effects, thereby impacting signal detection. Also, other patient-specific factors, such as the use of multiple antidepressant agents at varying doses, and the presence of a range of psychiatric comorbidities may have generally contributed to the variability of our efficacy data, and affected our ability to detect a treatment signal.

## Conclusions

This was clearly a negative clinical trial with respect to large treatment effects of quetiapine XR for SSRI-resistant PD. Low doses of quetiapine XR appeared to be well-tolerated in PD patients, noteworthy in a patient population that is generally fearful of medication changes and side-effects. Metabolic and extrapyramidal side-effects were minimal. Sleep benefits are a potential advantage of this augmentation strategy, which merit additional study.
